# Involvement of Actin-Regulating Factor Cofilin in the Inclusion Body Formation and RNA Synthesis of Human Parainfluenza Virus Type 3 via Interaction With the Nucleoprotein

**DOI:** 10.3389/fmicb.2019.00095

**Published:** 2019-02-01

**Authors:** Yinshuang Li, Chaoliang Zhang, Nan Lu, Xichuan Deng, Guangchao Zang, Shengwei Zhang, Hong Tang, Guangyuan Zhang

**Affiliations:** ^1^Pathogen Biology and Immunology Laboratory, and Laboratory of Tissue and Cell Biology, Experimental Teaching and Management Center, Chongqing Medical University, Chongqing, China; ^2^Department of the First Clinical Medicine, Chongqing Medical University, Chongqing, China; ^3^Department of Pathogen Biology, School of Basic Medicine, Chongqing Medical University, Chongqing, China; ^4^School of Laboratory Medicine, Hubei University of Chinese Medicine, Wuhan, China

**Keywords:** human parainfluenza virus type 3, nucleoprotein, cofilin, inclusion bodies, RNA synthesis

## Abstract

Human parainfluenza virus type 3 (HPIV3) is one of the primary pathogens that causing severe respiratory tract diseases in newborns and infants. It could induce inclusion bodies (IBs) in infected cells. Comprised of viral nucleoprotein (N) and phosphoprotein (P), as well as some cellular factors, HPIV3 IBs are unique platform for efficient viral synthesis. Although several studies have demonstrated the formation of IBs, little is known about cellular proteins involved in HPIV3 IBs formation. By quantitative real-time PCR assays after cytochalasin D treatment, we found actin microfilaments of the cytoskeleton were indispensible for HPIV3 RNA synthesis. Using co-immunoprecipitation and immunofluorescence assays, an actin-modulating protein, cofilin was found to involve in the IBs formation through interaction with the N protein in N–P induced IBs complex. Viral IBs formation reduced upon RNA interference knockdown of cellular cofilin, thus viral RNA synthesis and protein expression level were also suppressed. What’s more, the inactive form of cofilin, p-cofilin was increased after HPIV3 infection, and phosphorylation of cofilin was required for interacting with N–P complex and IBs formation. We further identified that the regions in cofilin interacting with N protein lies in the C-terminus. Our findings for the first time to state that cellular cofilin involves in HPIV3 IBs and interaction with N is critical for cofilin to aid IBs formation and enhancing viral RNA synthesis.

## Introduction

For human parainfluenza virus type 3 (HPIV3) belongs to the family Paramyxoviridae, order Mononegavirales, and is an enveloped virus with a non-segmented negative-strand (NNS) RNA genome. As one of the primary pathogens that cause severe respiratory tract diseases including bronchiolitis, pneumonia, and croup in infants and young children (Murphy et al., 1988), no valid antiviral therapy or vaccine is currently available for HPIV3. Thus, a more complete understanding of the cellular factors that influence HPIV3 replication and pathogenesis is therefore necessary to aid in the development of vaccines and anti-viral therapies.

The genome of HPIV3 is 15,462 nucleotides in length (Stokes et al., 1992), and encodes six main viral proteins: the nucleoprotein (N), phosphoprotein (P), RNA-dependent RNA polymerase large protein (L), matrix (M) protein, and two spike glycoproteins consisting of hemagglutinin-neuraminidase (HN) protein and fusion (F) protein (Spriggs and Collins, 1986; Galinski, 1991). HN is involved in viral attachment to the host cell, while F is required for fusion with the host cell plasma membrane. M protein binds directly to the inner face of viral envelope and is the main force driving viral assembly and budding (Zhang et al., 2014). In the center of spherical HPIV3 virion, lie the ribonucleo-protein (RNP) complex, including genome RNA, the N protein, P protein, and L protein (Moscona, 2005). The viral RNA is encapsidated by N protein to form N-RNA template, and RNA polymerase consisting of L protein and cofactor P protein associate with N-RNA template to form the active RNP complex necessary for transcription and replication.

As have been reported for a variety of NNS viruses, like human respiratory syncytial virus (Garcia et al., 1993), rabies virus (Lahaye et al., 2009), vesicular stomatitis virus (VSV) (Heinrich et al., 2010), and Ebola virus (Hoenen et al., 2012), HPIV3 could also induce inclusion bodies (IBs) which are recognized as the replication factories and are accumulated aggregates of viral proteins, as well as certain cellular proteins (Menager et al., 2009; Radhakrishnan et al., 2010; Koga et al., 2015). Previous study showed that the minimal protein requirement for HPIV3 IBs formation is N and P proteins (Zhang et al., 2013), and co-expression of N and P in mammalian cells could produce unique dot-like IBs and these highly dynamic structures fuse together to form larger functional IBs. Furthermore, a microtubule-related cell factor, acetylated α-tubulin was found to enhance viral replication by facilitating the fusion of HPIV3 IBs (Zhang et al., 2017). However, the cellular proteins involved in the formation of HPIV3 IBs are still largely unknown.

As one of the main cytoskeletal structure, actin filament (F-actin) has been shown plays essential roles in numerous viruses’ life cycle. For example, measles virus (Wakimoto et al., 2013), human immunodeficiency virus-1 (Wilk et al., 1999), influenza A virus (Smirnova et al., 2013), and herpes simplex virus (Mingo et al., 2012). Most importantly, intact F-actin was found essential to HPIV3 RNA replication and transcription (Gupta et al., 1998; De and Banerjee, 1999). Dynamic polymerization and depolymerization of F-actin are highly modified by a number of actin binding proteins, which cooperatively control the assembly and disassembly of F-actin (Pollard and Borisy, 2003; Pivovarova et al., 2013). Cofilin is a well-known regulator that responsible for remodeling the actin cytoskeleton and has two isoforms in mammalian cells: cofilin-1 (non-muscle cofilin, as referred in this paper) and cofilin 2 (muscle cofilin) (Bernstein and Bamburg, 2010). Cofilin binds and severs F-actin to induce depolymerization of F-actin and it is inactivated by phosphorylation of the serine residue at position 3, resulting in polymerization of F-actin (Arber et al., 1998).

In this study, we searched for cellular factors that participate in HPIV3 replication and transcription. An F-actin regulating protein, cofilin was found to play important role in HPIV3 IBs formation and viral RNA synthesis, further research showed that phosphorylation of cofilin is essential for its interaction and association with IBs complex. What’s more, by truncated mutation analysis, we preliminary identified that the regions that responsible for the interaction with N protein in N–P complex lie in the C-terminus in cofilin.

## Materials and Methods

### Cells and Viruses

293T, HeLa, A549, LLC-MK2 (MK2), and BHK-21 cells were cultured in Dulbecco’s modified Eagle’s medium (DMEM, Hyclone) supplemented with 16% fetal bovine serum (FBS, EveryGreen) and 1% penicillin–streptomycin. HPIV3 (NIH 47885), recombinant HPIV3_HA-P_ and VSV were kindly provided by Professor Mingzhou Chen of Wuhan University. HPIV3 and HPIV3_HA-P_ were propagated in MK2 cells by inoculation at an multiplicity of infection (MOI) of 0.1.

### Plasmid Constructs and siRNAs

The plasmids pCAGGS-N-Flag encoding HPIV3 N protein with a Flag tag in its C terminus, and pCAGGS-HA-P encoding P protein with a HA tag in its N terminus, were also provided by Professor Mingzhou Chen. To construct the plasmids encoding wt cofilin, mutant S3A (serine at position 3 substituted by alanine) and the truncated mutants cof△N20, cof△N40, cof△N83, cof△C20, cof△C40, and cof△C80, cDNAs encoding wild type cofilin, S3A and the above truncated mutants with Myc tag fused to their C terminus were amplified by PCR-based cloning techniques and cloned into expression plasmid pCAGGS-MCS. All constructs were verified by DNA sequencing. The small interfering RNAs (siRNAs) against cofilin (si-cofilin) consist of oligonucleotides with the sequences 5′-GUCUUCAACGCCAGAGGAGTT-3′ and 5′-CUCCUCUGGCGUUGAAGACTT-3′. The sequences of scrambled siRNAs (si-NC) were 5′-UUCUCCGAACGU GUCACGUTT-3′ and 5′-ACGUGACACGUUCGGAGAATT-3′.

### Western Blot Analysis

Infected or transfected cells were harvested and lysed in cold TNE buffer (50 mM Tris-Cl [pH 7.4], 150 mM NaCl, 2 mM EDTA [pH 8.0], 0.1% 2-mercaptoethanol and protease inhibitor cocktail). After incubation on ice for 30 min, cell lysates were centrifuged at 13,000 rpm for 30 min at 4°C. The clarified supernatant was mixed with 5XSDS-PAGE loading buffer, boiled at 100°C for 10 min and then subjected to 12% sodium dodecyl sulfate-polyacrylamide gel. The primary antibodies used were as follows: mouse anti-HPIV3 (1:2500, Abcam), rabbit anti-β-actin (1:1000, Proteintech), mouse anti-Myc tag (1:2500, Santa Cruz), mouse anti-HA tag (1:10000, Sigma), mouse anti-Flag tag (1:2500, Sigma), mouse anti-cofilin (1:2500, Proteintech), and rabbit anti-p-cofilin (1:1000, Cell Signaling Technology). HRP-conjugated goat anti-mouse IgG (1:5000, Sigma) and HRP-conjugated goat anti-rabbit IgG (1:5000, Sigma) were used as secondary antibodies.

### *In vitro* Co-immunoprecipitation

293T cells in 10 cm dishes were grown to 50–60% confluent and transfected with the indicated plasmids by calcium phosphate transfection reagent. At 48 h posttransfection, cells were harvested and lysed in 300 ul TNE buffer as described above. 50 ul of each lysates were mixed with SDS-PAGE loading buffer and boiled for input analysis, the rest lysates were incubated with anti-Myc antibody or anti-cofilin antibody for 1 h at 4°C with gentle rotation. After short centrifugation, samples were incubated with 40 ul of pretreated (washed once with TNE buffer) protein A+G Agarose Fast Flow medium at 4°C with gentle rotation overnight. Beads were then collected by short centrifugation at 8,000 rpm. After five times wash with washing buffer (5% sucrose, 5 mM Tris-Cl [pH 7.4], 5 mM EDTA [pH 8.0], 0.5 M NaCl, and 1% Triton X-100 [wt/vol]), bound proteins were eluted from beads by boiling with SDS-PAGE loading buffer, then analyzed by Western blot as described above.

### Immunofluorescence Assay

Hela or A549 cells were washed three times with cold PBS, then fixed with 4% paraformaldehyde for 20 min, permeabilized with 0.2 % Triton X-100 for 20 min. After blocking with 3% bovine serum albumin (BSA) in PBS for 30 min, cells were stained with relative primary antibodies for 1 h at room temperature. The primary antibodies used including mouse anti α*-*tubulin, rabbit anti-Flag tag, mouse anti-HA tag, rabbit anti-HA tag, mouse anti-Myc tag, mouse anti-cofilin antibodies depends on situation. The cells were then washed three times with 1% BSA and incubated with relative secondary antibodies for 45 min at room temperature. The secondary antibodies used were: Alexa Fluor 488-conjugated goat anti-rabbit IgG (1:1000, Thermo), Alexa Fluor 488-conjugated goat anti-mouse IgG (1:1000, Thermo) and Alexa Fluor 568-conjugated goat anti-mouse IgG (1:1000, Thermo). F-actin was stained by Alexa Fluor 488-conjugated Phalloidin (1:1000, AAT Bioquest), and cell nuclei were stained with DAPI (Solarbio) in some experiments. Images were observed via an Immunofluorescence microscope.

### Quantitative Real-Time PCR

Total RNA was extracted from Hela cells with RNA Extraction Kit (TIANGEN), treated with DNase I, and reverse transcribed into cDNAs using the M-MLV reverse transcriptase (Promega) and random hexamer primers. The quantities of HPIV3 N and P genes, VSV N and P genes, cellular cofilin, and β-actin were quantified using SYBR Premix Ex Taq II (Takara) and a LightCycler (Roche). Data shown are the relative abundance of the indicated genes normalized to that of β-actin. The primers were as follows: HPIV3 N gene forward: 5′-GTGGTTAAGACGAGA-GAGATG-3′, HPIV3 N gene reverse: 5′-GTCTGAAAGCCTCTAATCGAGT-3′, HPIV3 P gene forward: 5′-CCAAGAGATAAATCAACTAAT-3′, HPIV3 P gene reverse: 5′-TCAATATTTCTATCTTTTGC-3′, Human cofilin forward: 5′-ATGGCCTCCGGTGTGGCTGTCTCTG-3′, Human cofilin reverse: 5′-TCACAAAGGCTTGCCC-TCCAGGGAG-3′, VSV N gene forward: 5′-GGCAGAGATGTGG-TCGAATG-3′, VSV N gene reverse: 5′-CTCTCTTAGGCCTTGCAGTG-3′, VSV P gene forward: 5′-GATGAGATCGAAGCACAACG-3′, VSV P gene reverse: 5′-GCTTCTGGATCTGGTG-CATAC-3′.

### Virus Infection and Plaque Assay

Cells were grown to 60–70% confluent and infected with wild type HPIV3, HPIV3_HA-P_ or VSV for 1 h at 37°C and 5% CO_2_, then the infection medium was removed and changed with fresh medium containing 4% FBS. In the plaque assay, HPIV3-containing culture medium was serial 10-fold diluted up to 10^-5^, MK2 cells in 24-well plates grown to 60–70% confluent were infected with 400 ul of the dilutions. For VSV titer determination, VSV-containing culture medium was serial 10-fold diluted up to 10^-7^, BHK-21 cells in 24-well plates were infected. After incubation for 2 h at 37°C and 5% CO_2_, the infection medium was replaced with methylcellulose, and the cell plates were incubated at 37°C and 5% CO_2_ for another 3–4 days until visible viral plaques were detected. Plates were stained with 0.5% crystal violet for at least 4 h at room temperature and washed, then the plaques were countered and the viral titers were calculated.

## Results

### Disruption of the F-Actin Structure Specifically Affects HPIV3 RNA Synthesis

**FIGURE 1:**
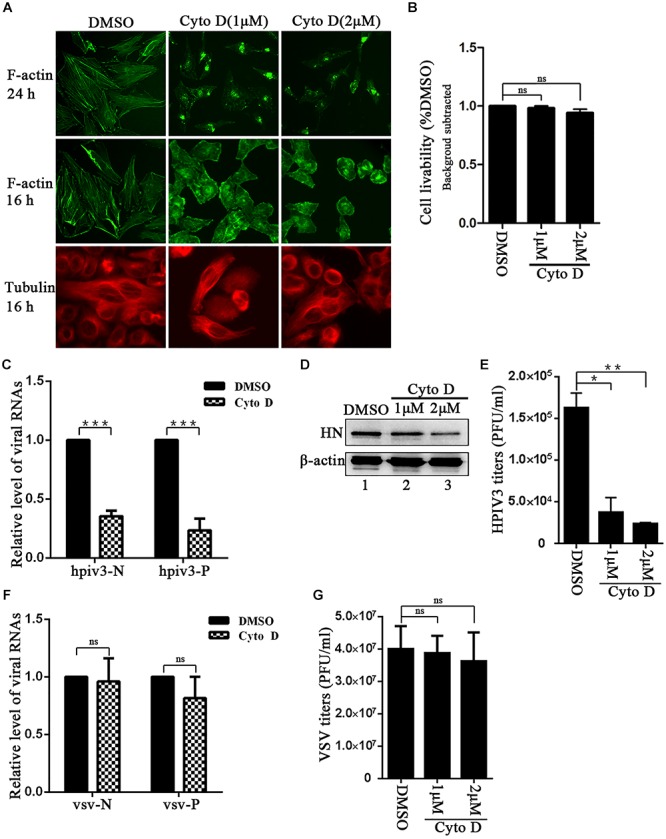
F-actin disruption specifically affects HPIV3 RNA synthesis. **(A)** Hela cells were treated with cyto D (1 μM or 2 μM) or control DMSO. F-actin structures were stained with phalloidin-AF488 at 24 h and 16 h after incubation, microtubules were stained with mouse anti-α-tubulin primary antibody and goat anti-mouse AF568 at 16 h after incubation as described in “Materials and Methods” section. Images were observed using an immunofluorescence (IF) microscope. **(B)** Hela cells incubated with cyto D or DMSO for 24 h, and the cell livability was examined by CCK8 assay as the manufacture’s instruction. There was no significant toxicity when Hela cells were treated with 1 μM or 2 μM cyto D. Student’s test: ns, non-significant. **(C)** Hela cells were infected with HPIV3(MOI = 1) for 8 h, then cyto D (2 μM) or DMSO were added, at 24 h postinfection, the cells were collected and real-time PCR was performed as described in “Materials and Methods” section to detect HPIV3 N and P RNAs. Cellular β-actin mRNA was used as control. Samples were examined in triplicate, and data are means ± SD from three experiments. Student’s test: ^∗∗∗^*p* < 0.001. **(D,E)** Hela cells were treated as above. At 24 h postinfection, cells were collected and viral protein was analyzed by Western blot (WB). Cellular β-actin was used as a loading control. Viral titers in the cell supernatant were determined by plaque assay as described in “Materials and Methods” section. Data are means ± SD from three experiments. Student’s test: ^∗^*p* < 0.05; ^∗∗^*p* < 0.01. **(F,G)** Hela cells were infected with VSV at an MOI of 0.5 for 8 h, and then cyto D or DMSO were added. At 24 h postinfection, the cells were collected and real-time PCR was performed as described in “Materials and Methods” section to detect VSV N and P RNAs. Cellular β-actin mRNA was used as control. Samples were examined in triplicate, and data are means ± SD from three experiments. Student’s test: ns, non-significant. Viral titers in the cell supernatant were determined. Data are means ± SD from three experiments. Student’s test: ns, non-significant.

To first visualize the effect of cyto D on F-actin structure, Hela cells were treated with 1 μM or 2 μM cyto D for 24 h, and immunofluorescence assays were conducted in which F-actin were stained. Immunofluorescence images showed that incubation with 1 μM or 2 μM cyto D for 24 h resulted in the disappearance of stress fibers and the actin-dence cortex, which were clearly observed in DMSO treated cells (Figure 1A, upper three images), indicating that cyto D indeed disrupted the intact network of F-actin. However, a substantial contraction of cytoplasm was shown at 24 h treatment. In order to exclude the unintended effect of treatment to cytoskeleton and to test the specificity of the cyto D treatment, cells were treated with 1 μM or 2 μM cyto D for 16 h, and then either F-actin or microtubules were stained. The results showed that cytoplasm was slightly contracted by a shorter time treatment, but F-actin fiber structure still disappeared as before (Figure 1A, middle three images). In contrast, the stress fiber structure of microtubules kept unaffected upon the cyto D treatment (Figure 1A, bottom three images), indicating that cyto D specifically disrupt cellular F-actin structures. To exclude the potential toxicity of cyto D to cells, cytotoxicity assays were performed. After treated with the indicated concentration of cyto D for 24 h, the viability of the cells were examined by CCK-8 assays, and the results showed that there was no significant cytotoxic effects to cells upon cyto D treatment (Figure 1B). In order to determine the role that F-actin plays in HPIV3 life cycle, Hela cells were infected with HPIV3 for 8 h, then treated with either cyto D or mock DMSO for another 16 h. After HPIV3 infection and cyto D treatment, cells were harvested and proceeded real-time-PCR and Western blot assays. The results showed that both the viral RNA synthesis and protein expression level were suppressed by cyto D treatment, since HPIV3 N and P RNAs, as well as expression of viral HN proteins were significantly decreased compared to DMSO treatment (Figures 1C,D, upper blot). Whereas the level of cellular β-actin maintained stable, since cyto D only binds to F-actin polymer and prevents polymerization of actin monomers (Figure 1D, lower blot). As a result, viral titers in the supernatants from cyto D treated cells were also notably reduced, compared to DMSO-treated controls (Figure 1E). In order to show RNA synthesis and virus titer reduction by the cyto D treatment is HPIV3 specific, VSV, which is also enveloped NNS RNA virus was used as control. The results showed that the same treatment with cyto D inhibit neither the RNA synthesis nor the virus titer of VSV. The effect of cyto D on VSV N and P RNAs were not significant compared to DMSO control (Figure 1F), and VSV titers were also comparable between cyto D and DMSO treatment (Figure 1G). These results indicate that intact F-actin structures were specifically crucial to HPIV3 RNA synthesis.

### Cofilin Associates With the N–P Induced IBs

**FIGURE 2:**
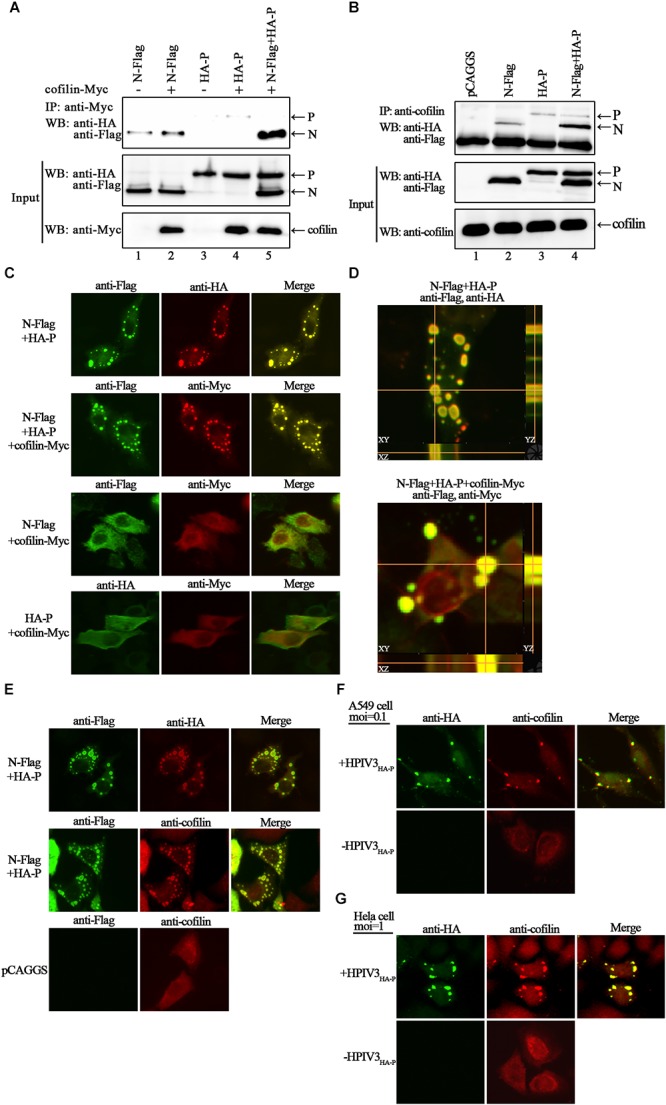
Cofilin interacts with N protein in N–P complex and colocalizes with N–P induced IBs. **(A)** 293T cells were transfected with plasmids encoding N-Flag, HA-P and cofilin-Myc individually or jointly as indicated. At 48 h posttransfection, the cells were collected and processed to co-IP assays as described in “Materials and Methods” section. Proteins were precipitated with anti-Myc antibody and examined by WB. **(B)** 293T cells were transfected with the indicated plasmids. At 48 h posttransfection, co-IP assays were performed as described above. Proteins were precipitated with anti-cofilin antibody and examined by WB. **(C,D)** Hela cells were cotransfected with plasmids expressing N-Flag, HA-P and cofilin-Myc as indicated by lipo3000 reagent as the manufacture’s instruction. At 24 h posttransfection, the cells were fixed, and rabbit Flag primary antibody and goat anti-rabbit AF488 were used to stain N, mouse HA primary antibody and goat anti-mouse AF568 were used to stain P (C, upper, middle image), rabbit HA primary antibody and goat anti-rabbit AF488 were used to stain P (C, bottom, left image), and mouse Myc primary antibody and goat anti-mouse AF568 were used to stain cofilin. Z-stack images for the colocalization of N and P (**D**, upper image), N–P induced IBs and cofilin (**D**, bottom image). Images were analyzed via IF microscope. **(E)** Hela cells were cotransfected with plasmids expressing N-Flag and HA-P or mock transfected with pCAGGS for 24 h. the cells were fixed, and rabbit Flag primary antibody and goat anti-rabbit AF488 were used to stain N, mouse HA primary antibody and goat anti-mouse AF568 were used to stain P, mouse cofilin primary antibody and goat anti-mouse AF568 were used to stain cellular cofilin. **(F,G)** A549 or Hela cells were infected with HPIV3_HA-P_ at an MOI of 0.1 or 1, or mock infected. At 24 h postinfection, cells were fixed and rabbit HA primary antibody and goat anti-rabbit AF488 were used to stain viral IBs, mouse cofilin primary antibody and goat anti-mouse AF568 were used to stain cellular cofilin. Images were analyzed via IF microscope.

To search for certain proteins related to the transcription and replication process of HPIV3, we focused on cofilin, which is a main regulator of actin cytoskeleton reorganization and has been found involving in the formation of measles virus ribonucleoprotein complex (Koga et al., 2015). Firstly, we constructed a plasmid encoding Myc-tagged cofilin and examined the interaction between exogenous cofilin-Myc and HPIV3 N–P complex via co-immunoprecipitation assays. The results showed that when cofilin-Myc was transiently co-expressed with N or P protein and co-IP assays were performed by precipitating cofilin-Myc, only a small amount of N or P proteins were co-precipitated (Figure 2A, upper blot, lanes 2 and 4), indicating that Myc-tagged cofilin only slightly interact with either single N or P. However, when N and P were co-expressed to form the N–P complex, the interaction between cofilin-Myc and N protein was greatly increased but the interaction between cofilin-Myc and P protein was obviously decreased (Figure 2A, upper blot, lane 5). What’s more, similar co-IP assays were performed in which endogenous cofilin were precipitated. Compared with the slight interaction with N or P protein alone (Figure 2B, upper blot, lane 2 and 3), endogenous cofilin strongly interacted with N protein when P was also present, and more associate with N protein may result in poorer cofilin to interact with P protein in the N–P complex (Figure 2B, upper blot, lane 4). These data above indicate that cellular cofilin mainly associates with the N protein in the N–P complex.

Next, we examined the intracellular distribution of cofilin when co-expressed with N or/and P proteins by immunofluorescence assays. Co-expression of N and P proteins formed the dot-like IBs as have been reported previously. Exogenous myc-tagged cofilin sufficiently co-localized with N–P induced IBs when co-expressed with N and P proteins. However, when cofilin-Myc was co-expressed with single N or P, the dot-like IBs were disappear, all the proteins were present throughout the cells and co-localized partially (Figure 2C). To further validate the colocalization of cofilin with N–P induced IBs, Z-stack of the images was performed. Orthogonal views of the XY, XZ, and YZ images confirmed the three-dimensional colocalization of cofilin and IBs (Figure 2D). Meanwhile, unlike individually dispersed distribution, endogenous cofilin also re-localized with dot-like IBs in the presence of N and P proteins (Figure 2E). To determine whether endogenous cofilin co-localized with viral IBs in HPIV3-infected cells, a recombinant HPIV3 virus with a HA tag fused to the N terminus of P, i.e., HPIV3_HA-P_ was used. As expected, cellular cofilin also co-localized with IBs in recombinant HPIV3_HA-P_ infected A549 and Hela cells (Figures 2F,G), quite different from those in uninfected cells. These results correlate with above co-IP assays and indicate that cofilin mainly interacts with the N protein in the N–P complex and co-localize with viral IBs.

### Knockdown of Cofilin Results in Decrease of IBs Formation and Viral RNA Synthesis

**FIGURE 3:**
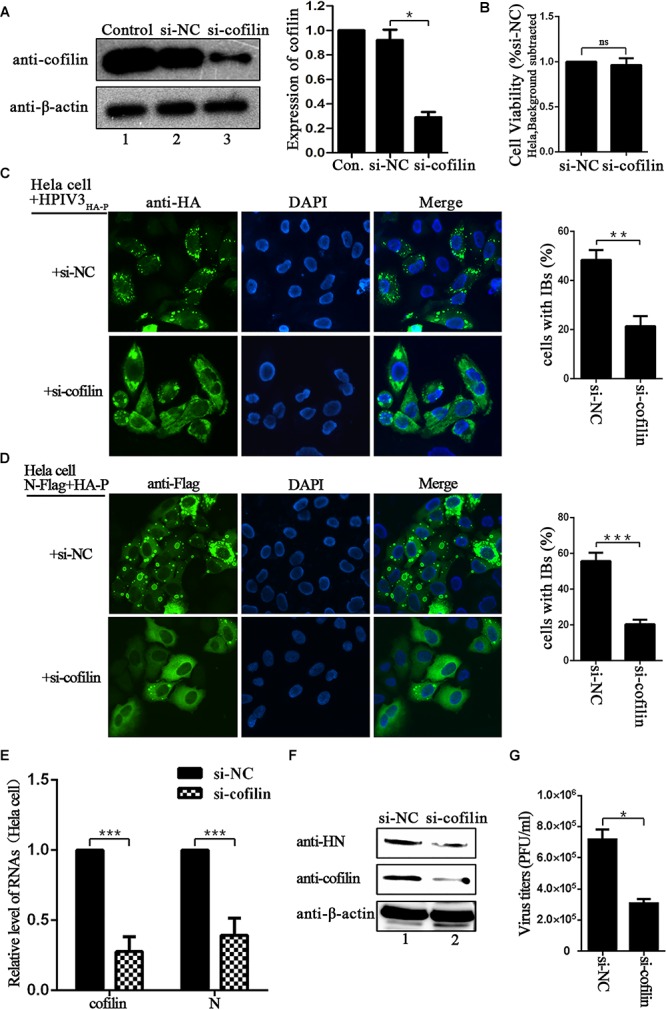
Depletion of cofilin affects IBs formation and RNA synthesis in Hela cells. **(A)** Hela cells were mock transfected or transfected with si-NC control or si-cofilin targeting cellular cofilin. Expression level of cofilin was analyzed by WB. Cellular β-actin was used as a loading control. Data are means ± SD from three experiments. Student’s test: ^∗^*p* < 0.05. **(B)** Hela cells transfected with si-NC or si-cofilin for 24 h, and the cell viability was examined by CCK8 assay as the manufacture’s instruction. The viability of cell was not significantly influenced by cofilin depletion. Data are means ± SD from three experiments. Student’s test: ns, non-significant. **(C)** Hela cells were transfected with si-NC or si-cofilin, and infected with HPIV3_HA-P_ at an MOI of 1 after 24 h transfection. At 24 h postinfection, the cells were fixed, HA-P was immunostained to visualize IBs and nuclei were counterstained with DAPI. The percentage of cells containing IBs were quantified, data are means ± SD from three experiments. Student’s test: ^∗∗^*p* < 0.01. **(D)** Hela cells were transfected with the plasmids encoding N-Flag and HA-P, as well as si-NC or si-cofilin. At 24 h posttransfection, the cells were fixed, N-Flag was immunostained to visualize IBs and nuclei were counterstained with DAPI. The percentage of cells containing IBs were quantified, data are means ± SD from three experiments. Student’s test: ^∗∗∗^*p* < 0.001. **(E)** Hela cells were transfected with si-NC or si-cofilin, and infected with wild type HPIV3 at an MOI of 1 after 24 h transfection. At 24 h postinfection, the cells were collected and real-time PCR was performed to detect RNAs of cofilin and N. Cellular β-actin mRNA was used as the control. Samples were examined in triplicate, and data are means ± SD from three experiments. Student’s test: ^∗∗∗^*p* < 0.001. **(F,G)** Hela cells were transfected and infected the same as described in the legend for **(E),** then the cells were collected and viral HN protein and cellular cofilin were analyzed by Western blot (WB). Cellular β-actin was used as a loading control. Viral titers in the cell supernatant were determined by plaque assay as described in “Materials and Methods” section. Data are means ± SD from three experiments. Student’s test: ^∗^*p* < 0.05.

Since IBs are rendered as the replication factories of HPIV3, we sought to determine the potential role for cofilin including in this process. To test the effect of cofilin knockdown on IBs formation, Hela cells were transfected with either control si-NC or si-cofilin targeting human cofilin. Western blot assays showed that the efficiency of cofilin depletion was at least 50% (Figure 3A). CCK-8 assays showed that Cofilin knockdown did not influence the viability of Hela cells (Figure 3B). Then the cells were infected with HPIV3_HA-P_ after si-cofilin transfection, the results of immunofluorescence assays showed that knockdown of cofilin greatly affected the level of viral IBs formation in HPIV3_HA-P_ infected cells, and the number of cells containing dot-like IBs reduced about 30% compared to that of si-NC transfected cells (Figure 3C). It was the same case for IBs formation in the cells co-transfected with N and P proteins, together with si-cofilin, the number of cells containing N–P induced IBs reduced at least 30% compared to that of si-NC transfected cells (Figure 3D). Furthermore, we examined the level of viral RNA synthesis upon siRNA-mediated knockdown of cellular cofilin. Hela cells were transfected with si-NC or si-cofilin, and infected with wild-type HPIV3, then real-time-PCR assays were performed. The results showed that when the level of cellular cofilin reduced to 30%, viral N RNAs were significantly suppressed and reduced to about 40% by cofilin knockdown compared with si-NC control (Figure 3E). What’s more, protein expression level was also affected by knockdown of cofilin, since viral HN proteins greatly decreased in si-cofilin transfected cells (Figure 3F). As a result of lower RNA synthesis and protein expression level, viral titers significantly reduced in si-cofilin transfected culture supernatant compared to that of si-NC control (Figure 3G).

**FIGURE 4:**
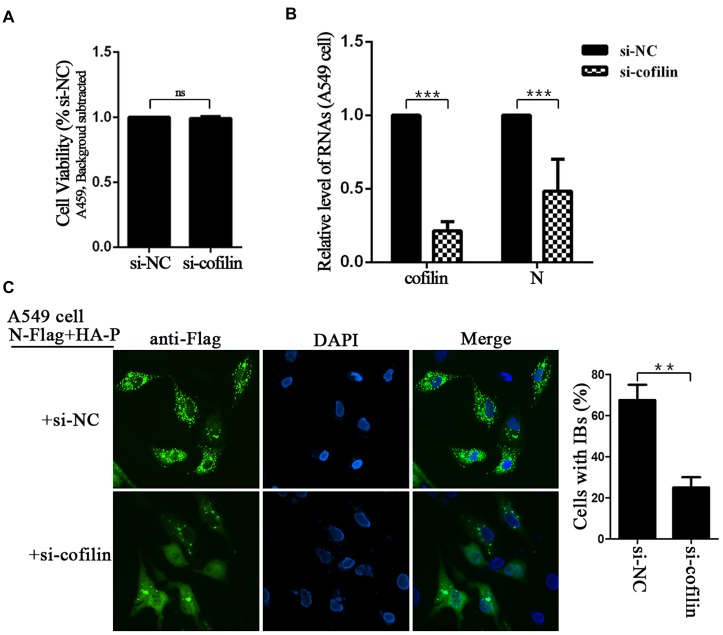
Depletion of cofilin affects IBs formation and RNA synthesis in A549 cells. **(A)** A549 cells transfected with si-NC or si-cofilin for 24 h, and the cell viability was examined by CCK8 assay as the manufacture’s instruction. The viability of cell was not significantly influenced by cofilin depletion. Data are means ± SD from three experiments. Student’s test: ns, non-significant. **(B)** A549 cells were infected with wild type HPIV3 at an MOI of 1 for 6 h, then si-NC or si-cofilin were transfected. At 24 h posttransfection, the cells were collected and real-time PCR was performed to detect RNAs of cofilin and N. Cellular β-actin mRNA was used as the control. Samples were examined in triplicate, and data are means ± SD from three experiments. Student’s test: ^∗∗∗^*p* < 0.001. **(C)** A549 cells were transfected with the plasmids encoding N-Flag and HA-P, as well as si-NC or si-cofilin. At 24 h posttransfection, the cells were fixed, N-Flag was immunostained to visualize IBs and nuclei were counterstained with DAPI. The percentage of cells containing IBs were quantified, data are means ± SD from three experiments. Student’s test: ^∗∗^*p* < 0.05.

The effect of cofilin-depletion on IBs and HPIV3 RNA synthesis was also determined in A549 cells. CCK-8 assays showed that cofilin knockdown did not influence the viability of A549 cells (Figure 4A). To exclude that cofilin knockdown may impact HPIV3 entry into the cells, thus has an effect on viral RNA synthesis, A549 cells were first infected with wild type HPIV3 for 6 h to allow entry of the virus, then si-NC or si-cofilin was transfected. At 24 h after transfection, the cells were harvested and qRT-PCR assays were performed to examine viral RNA synthesis. The results showed that viral N RNAs were still greatly suppressed and reduced to no more than 50% by cofilin depletion compared with si-NC control (Figure 4B). The data was similar with the results obtained in Hela cells and indicated that cofilin knockdown reduced HPIV3 RNA synthesis in A549 cells. As expected, when N and P proteins, together with si-cofilin were cotransfected into A549 cells, N–P induced IBs were also significantly decreased, the number of cells containing IBs reduced at least 40% compared to that of si-NC transfected cells (Figure 4C). These results above indicate that involvement of cofilin in IBs formation is important for viral RNA synthesis.

### HPIV3 Induced Phosphorylation Is Important for Cofilin to Interact With N–P Complex and Aid IBs Formation

**FIGURE 5:**
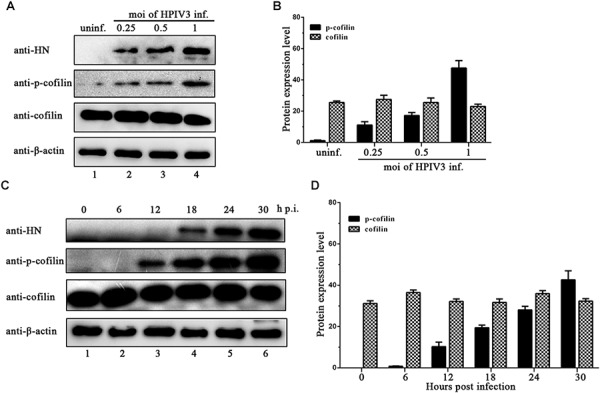
HPIV3 infection induces cofilin phosphorylation. **(A,B)** Hela cells were mock infected or infected with HPIV3 at MOI of 0.25, 0.5, and 1, respectively. At 24 h after infection, the cells were collected and subjected to WB to determine the protein expression level of viral HN, cellular cofilin, p-cofilin and β-actin. Relative expression level of cofilin and p-cofilin were calculated by normalized to that of β-actin. **(C,D)** Hela cells were infected with HPIV3 at MOI of 1 and cultured for 0, 6, 12, 18, 24, 30 h. At different time points, cells were collected and subjected to WB as described in the legend of **(A,B)**.

**FIGURE 6:**
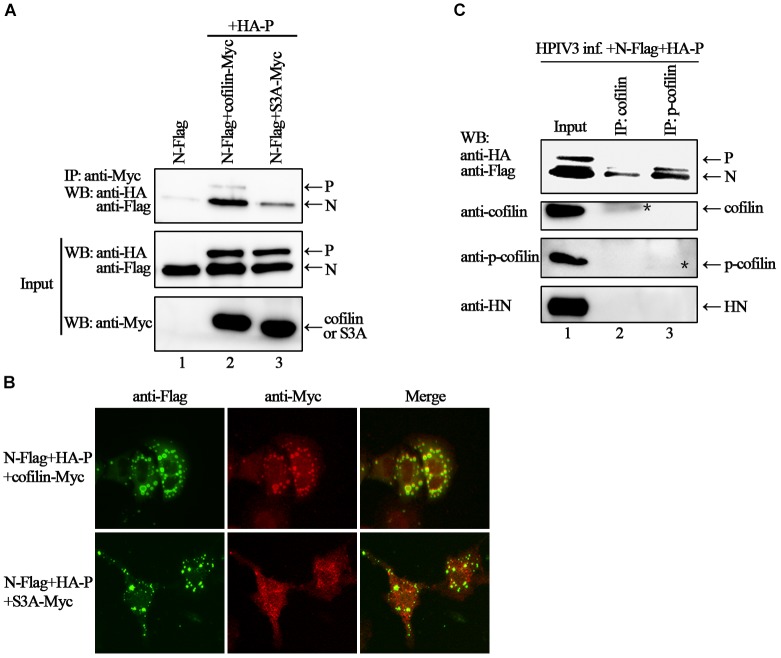
Phosphorylation is required for cofilin to associate and colocalize with N–P complex. **(A)** 293T cells were transfected with plasmids encoding N-Flag, HA-P, cofilin-Myc and S3A-Myc individually or jointly as indicated. At 48 h after transfection, the cells were collected and processed to co-IP assays as described in the legend of Figure 2A. Proteins were precipitated with anti-Myc antibody and examined by WB. **(B)** Hela cells were transfected with plasmids encoding N-Flag, HA-P and cofilin-Myc or S3A-Myc. At 24 h after transfection, the cells were fixed, and rabbit Flag primary antibody and goat anti-rabbit AF488 were used to stain N, and mouse Myc primary antibody and goat anti-mouse AF568 were used to stain cofilin. Images were analyzed via IF microscope. **(C)** Hela cells were infected with HPIV3 at MOI of 1 for 1 h, and then plasmids encoding N-Flag and HA-P were transfected. At 24 h posttransfection, the cells were collected and processed to co-IP assays as described in “Materials and Methods” section. Proteins were precipitated with anti-cofilin or p-cofilin antibodies and examined by WB. The stars indicated the bands of precipitated cofilin or p-cofilin.

Phosphorylation of serine residue at position 3 regulates the activity of cofilin. We then examined the expression levels of cofilin and phosphorylated form of cofilin, i.e., p-cofilin in HPIV3 infected cells. The results showed that during HPIV3 infection, total expression level of cofilin maintained relatively stable, however, the level of p-cofilin increased markedly (Figures 5A–D). These indicate that cofilin is inactivated during HPIV3 infection. Next, we analyzed the interaction of constitutively non-phosphorylated cofilin (S3A) with the N–P complex. The results of co-IP assays showed that when myc-tagged S3A were precipitated, fewer N and P were co-precipitated, compared with the results when cofilin-Myc were precipitated (Figure 6A, upper blot, lanes 2 and 3). Immunofluorescence assays showed that when co-transfected with N and P, cofilin-Myc co-localized with dot-like IBs as before, in contrast, mutant S3A were dispersed throughout the cells and could no longer co-localized in N–P induced IBs (Figure 6B). Furthermore, co-IP assays were performed in the HPIV3 infection and the co-expression of N and P proteins. When cellular cofilin and p-cofilin were precipitated, respectively, N proteins precipitated by p-cofilin were more than that of non-phosphorylated cofilin, indicating that during HPIV3 infection, N proteins in RNP complex tend to interact with phosphorylated cofilin (Figure 6C). Taken together, these results indicate that HPIV3 infection induces upregulation of p-cofilin, which is important for cofilin interaction with N–P complex and benefits IBs formation.

### The C-Terminus of Cofilin Is Indispensible for the Interaction With N Protein

**FIGURE 7:**
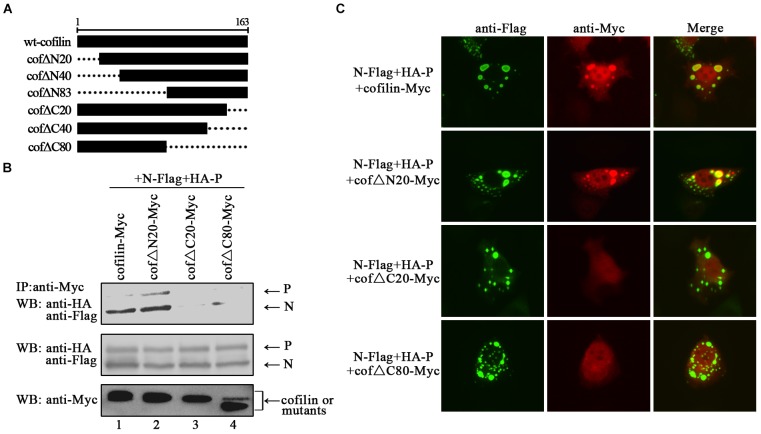
The C-terminus of cofilin is indispensible for the interaction with N protein. **(A)** Schematic of truncated mutants of cofilin. **(B)** 293T cells were cotransfected with plasmids encoding N-Flag, HA-P, and wild type or mutant cofilin as indicated. At 48 h after transfection, the cells were collected and processed to co-IP assays as described in the legend of Figure 2A. Proteins were precipitated with anti-Myc antibody and examined by WB. **(C)** Hela cells were transfected with plasmids encoding N-Flag, HA-P and wild type or mutant cofilin as indicated. At 24 h after transfection, the cells were fixed, and rabbit Flag primary antibody and goat anti-rabbit AF488 were used to stain N, and mouse Myc primary antibody and goat anti-mouse AF568 were used to stain wild type and mutant cofilin. Images were analyzed via IF microscope.

In order to identify the regions in cofilin that interact with N proteins, we constructed truncated mutants of cofilin, including N-terminus mutants cof△N20, cof△N40, and cof△N83, C-terminus mutants cof△C20, cof△C40, and cof△C80 (Figure 7A). However, cof△N40, cof△N83 and cof△C40 were not expressed in the cell lysates (data not shown), only cof△N20, cof△C20, and cof△C80 can be detected. Then the interactions between N protein and cof△N20, cof△C20, cof△C80 were examined via co-IP assays, respectively. The results showed that N proteins co-precipitated by cof△N20 were comparable to that of wild type cofilin, but fewer N proteins were co-precipitated by cof△C20 and cof△C80 (Figure 7B). Further immunofluorescence assays indicated that cof△C20 and cof△C80 were no longer co-localized with N–P induced IBs, and cof△N20 still colocalized in the IBs as wild type cofilin (Figure 7C), which consistant with the results of coIP. In conclusion, our results preliminary identified the regions that interact with N protein lies in the C-terminus within cofilin.

## Discussion

In the present study, we provide the evidence that cellular F-actin structures were specifically and crucial to HPIV3 RNA synthesis (Figure 1). The major finding of our study is that F-actin remodeling factor, cofilin associated and co-localized with viral IBs complex via direct interaction with the N protein (Figure 2) and the C terminus of cofilin is indispensible for the interaction (Figure 7). Moreover, cofilin is important for HPIV3 IBs formation and RNA synthesis, since knockdown of cofilin resulted in reduction of both IBs formation and viral RNA synthesis, as well as virus titers (Figures 3, 4). Further study showed that phosphorylation of cofilin, which induced by HPIV3 infection, was required for cofilin to interact with N–P complex and to aid IBs formation (Figures 5, 6).

Paramyxoviruses have been shown to utilize the cytoskeleton network for the completion of various activities in their life cycles (Cervera et al., 1981; Stallcup et al., 1983; Hamaguchi et al., 1985; Bohn et al., 1986), such as viral genome replication, assembly and release of progeny virions, and protein synthesis, though different viruses employ different host-virus interactions. In the HPIV3 system, the two cytoskeletal networks, actin microfilament and microtubule were both reported play important roles, respectively. Disruption of the microtubule by nocodazole inhibits HPIV3 release (Bose et al., 2001); acetylated microtubules regulate the fusion and maturation of HPIV3 IBs and enhance viral RNA synthesis (Zhang et al., 2017). Our results in this report indicate that F-actin play essential role in HPIV3 transcription and replication (Figure 1C), which are in line with the previous studies: actin microfilaments were the site for RNA synthesis, and treatment of the cells with cytochalasin D resulted in the inhibition of viral RNA synthesis and ribonucleoprotein accumulation in cells (Gupta et al., 1998).

Since the simultaneous addition of HPIV3 and cyto D to cells results in the loss of intracellular viral proteins (Figure 1D), which will consequently cause decrease of viral proteins released from cells, thus it is difficult for us to directly study the effect of F-actin on HPIV3 budding despite that HPIV3 titers were indeed affected by F-actin disruption in our study (Figure 1E). While we utilized the VLPs production system and examined M protein-mediated VLPs released from the cells after cyto D treatment. The results showed that, M VLPs production in the cyto D-treated cell supernatant was inhibited dramatically by disruption of F-actin compared with DMSO control, but the expression level of M protein in the whole cell lysates remained relatively stable (data not shown). Thus it suggests that F-actin might also required in the later stage of HPIV3 life cycle, for example, assembly and budding, since M proteins are believed to be adapters that link together the structural components of virions and drive their assembly. Moreover, a recent study suggests that RSV M protein interacts with F-actin, and M-actin interaction may play important role in transporting RSV RNPs to plasma membrane assembly site (Shahriari et al., 2018).

Here, we focused on the cellular factor cofilin, which plays an essential role in F-actin remodeling. We found that cofilin co-localized in viral IBs through main interaction with the HPIV3 N protein (Figure 2). Notably, cofilin could interact with single N or P only partially, while in the presence of both N and P, N seems to be the main target for cofilin to associate and relocalize in N–P induced IBs. Similar findings have been reported in measles virus that cellular cofilin interacts with the N protein and aids in the formation of the RNP complex (Koga et al., 2015). Meanwhile, we cannot exclude the possibility that in the N–P induced IBs complex, conformation of cofilin was changed by N or/and P and it tent to associate with N protein, thus blocks the sites for P-cofilin interaction. Our additional structure-function analysis of cofilin mutants revealed that cofilin C-terminal regions are critical for the N-cofilin interaction. Previous study reported that the C-terminal residues of actin form an extensive contact with the loop 41–46 and the N terminus of cofilin (Galkin et al., 2011). Our findings in this report suggest that the C terminus of cofilin is the regions that associate with HPIV3 N proteins (Figure 7), and this finding may yet reveal a novel mechanism that could be applicable to other enveloped viruses. Further results indicated that knockdown of cofilin, which should affect reassembly of F-actin, decreased IBs formation as well as viral mRNA level. Therefore, the involvement of cofilin in IBs formation suggests that HPIV3 may utilize F-actin for RNA synthesis indirectly, cellular cofilin works as a bridge between F-actin and viral IBs, and N protein may recognize and anchor the RNPs in F-actin through capturing its binding-protein cofilin. As one of the main cytoskeletal network, F-actin is also responsible for intracellular transport of organelles. Hence, when reorganization of F-actin is affected by depletion of cofilin, it is likely that transport of other putative host factors involved in HPIV3 IBs formation are influenced, as a result, RNA synthesis is influenced indirectly. What’s more, our previous studies have demonstrated that HPIV3 viral synthesis only occurs in large functional IBs, which derived from fusion and maturation of small IBs (Zhang et al., 2017), it is tempting to speculate that F-actin may offer platforms for IBs fusion, and disorganization of F-actin inhibits fusion of small IBs to form large functional IBs, thus RNA synthesis would be affected by the failure of IBs fusion. The detail mechanisms still remain to be discovered.

Moreover, our study demonstrated for the first time that HPIV3 infection resulted in upregulation of phosphorylated cofilin, which is enzymatically inactive, and this activity-decreased cofilin should result in enhanced F-actin polymerization. The constitutively non-phosphorylated mutant of cofilin, S3A did not co-localize with IBs any longer as a result of the loss of interaction with the N protein (Figure 5). As is well known that in the later stage of virus life cycle, as well as HPIV3, certain cytoskeletal frames or host factors are required for the transport of M protein and nucleocapsids to the sites of plasma membrane for budding. Although no evidences have been reported for actin filament in this process of HPIV3 so far, it has been reported in measles virus that stable actin filaments are needed for intracellular trafficking of viral RNPs to the plasma membrane (Dietzel et al., 2013), and F-actin in association with the M protein alters the interaction between the M and H proteins, thereby modulating measles virus cell-cell fusion and assembly (Wakimoto et al., 2013). Thus, it suggests that HPIV3 induced phosphorylation of cofilin may also contribute to later viral budding. Above all, it can be speculated that in HPIV3 infected cells, the N protein may sequester cellular cofilin in order to make viral IBs get access to the F-actin scaffold and carry out its RNA synthesis, then the virus reorganized actin cytoskeleton by phosphorylating cofilin, resulting in an increase of F-actin polymerization, which is more beneficial to RNA synthesis. While the detail mechanisms about how cofilin phosphorylation is induced by HPIV3 and whether HPIV3 infection may lead to activation of the phosphoinositide 3-kinase (PI3K) signaling pathway (Nebl et al., 2004). These questions remain uncovered and additional experiments will be needed to answer these questions further.

## Conclusion

In summary, our studies present the first investigation of cellular cofilin in interacting with HPIV3 N protein through its C-terminus and the interaction facilitates viral IBs formation, as well as viral RNA synthesis. We demonstrated further that HPIV3 induced phosphorylation is essential for cofilin to associate with N protein and to localize in IBs. Our findings contribute to progress in understanding virus–host relationship of HPIV3 and the N-cofilin interaction may be designated as valuable target for rational antiviral approaches. Future studies will focus on clarifying the detail mechanisms about the involvement of cofilin in HPIV3 IBs formation.

## Author Contributions

GyZ conceived, designed, and supervised the research and wrote original draft of the manuscript. YL, CZ, NL, and XD conducted the experiments. GyZ, NL, GcZ, and HT analyzed and interpreted the data. SZ contributed to discussion and provide important advice. All authors read and approved the final version of the manuscript.

## Conflict of Interest Statement

The authors declare that the research was conducted in the absence of any commercial or financial relationships that could be construed as a potential conflict of interest.
